# Integrative analysis identifies three molecular subsets in ovarian cancer

**DOI:** 10.1002/ctm2.1029

**Published:** 2022-09-18

**Authors:** Bo Liu, Xinchan Ji, Jinmeng Li, Nian Zhu, Junqi Long, Xujie Zhuang, Huina Wang, Lujia Li, Yuhaoran Chen, Shuangtao Zhao

**Affiliations:** ^1^ School of Software Engineering Faculty of Information Technology Beijing University of Technology Beijing 100124 China; ^2^ School of Mathematical and Computational Sciences Massey University Palmerston North 4472 New Zealand; ^3^ Department of Thoracic Surgery Beijing Tuberculosis and Thoracic Tumor Research Institute/Beijing Chest Hospital Capital Medical University Beijing 101149 China

Dear Editor,

We identified three novel molecular subtypes (tumour‐enriched, mixed and immune‐enriched) of ovarian cancer (OC) and revealed a complex molecular landscape among three subtypes. Several researches have studied the molecular subtypes of OC,^1^ yet more well‐recognized subtypes and more comprehensive analysis are needed. In our study, a multi‐platform analysis was implemented.

To profile the global genomic patterns of OC, a total of 4,188 tumour‐specific genes (Figure S1) were screened in 376 patients from The Cancer Genome Atlas, Gene Expression Omnibus and The Human Protein Atlas datasets (Figure 1A). Then, an unsupervised hierarchical clustering analysis was performed to reveal three subgroups in these OC patients (Figure 1B). Analysis of the canonical markers for lineage‐specific different expression genes (DEGs) suggested different tumour cell compositions among the three subgroups. OC patients of cluster A showed the highest expression of OC‐associated oncogenes *KRT16* and *KRT23*,^2^ and the lowest expression of immune cell markers *PTPRC* and *PDCD1*, suggesting enrichment of tumor cells. While OC samples of cluster C displayed the highest expression of the immune cell markers *PTPRC*, *PDCD1*, *HAVCR2* and *CD274* ^3^ and exhibited low or no expression of the cancer‐related oncogenes (*TBX21* and *NOTCH3* ^4^), indicating predominance of immune cells. And cluster B presented mixed expression characteristics of both cancer and immune cells, and was therefore classified as ‘mixed group’. We further examined the clinical factors of genomics‐based classification. Cluster A had significantly higher aneuploidy score and genome altered fraction than cluster C (*p* < .001), but cluster A and B had significantly lower diagnosis age and mutation count (*p* < .05, Figure 1B and Tables S1 and S2). Additionally, three groups have similarities on race, figo‐stage and hypoxia‐score. We also observed a trend of shortened survival in cluster A patients, although it did not reach a statistical significance in this study (log‐rank *p* = .500, Figure 1C, Figure S2 and Table S3). These results suggested similarities and differences in clinical factors, which can help with diagnosis and treatment.

**FIGURE 1:**
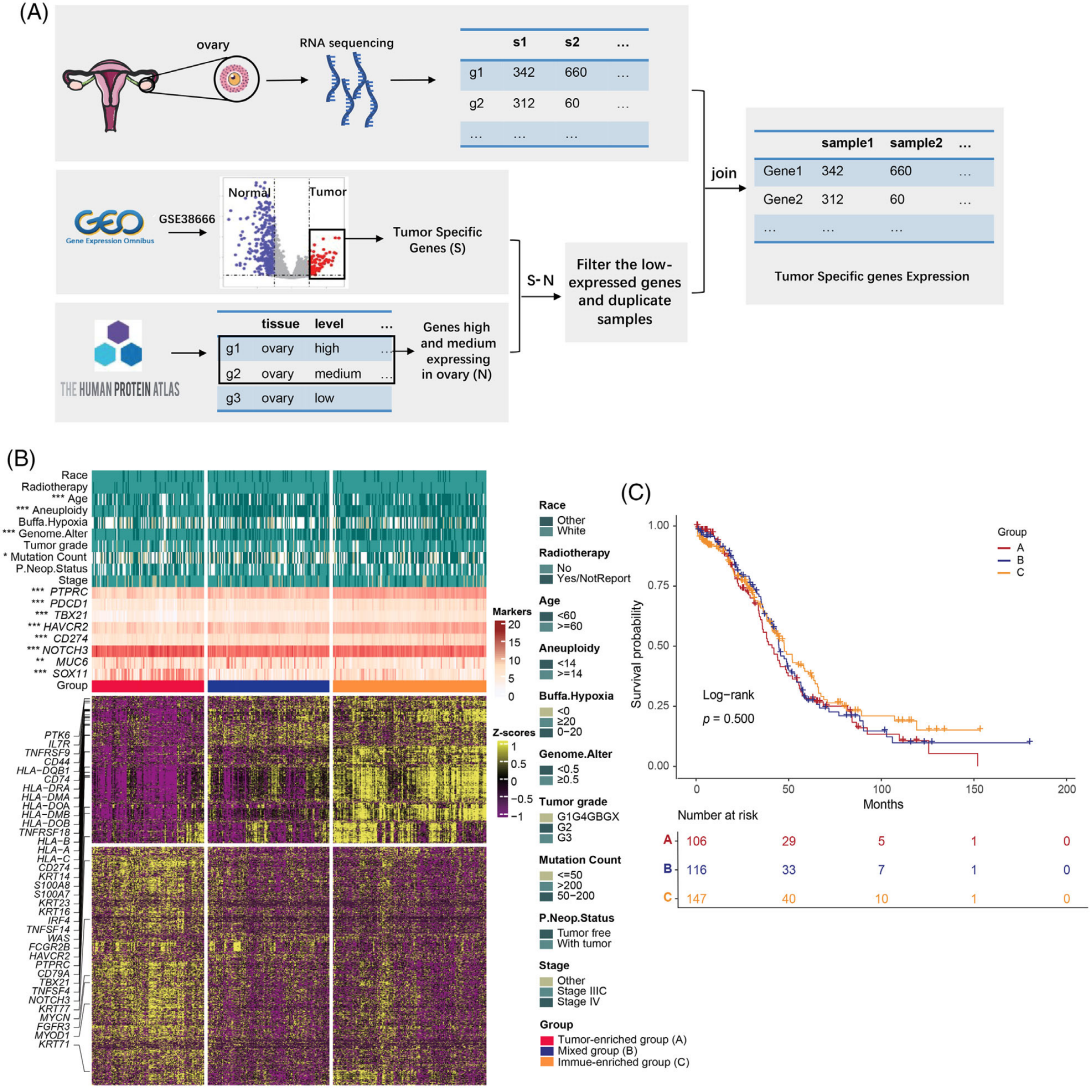
Genomic characterization defined three subgroups with different tumor and immune compositions. (A) Flow chart of the filtering processes enrolled into this study. (B) Genomic profiling of three clusters based on tumor and immune‐related genes expression. (C) Survival analysis among three subtypes.

Pathways enrichment analysis was performed in cluster A to explore the dysregulated molecular processes informed by the genomic data. A total of six up‐regulated KEGG: Kyoto Encyclopedia of Genes and Genomes pathways were screened, which mainly included oncogenic signallings (Figure 2A) such as WNT and MAPK signaling pathway, highlighting the importance of targeting the WNT and MAPK pathway as interested therapeutic strategies. Next, 777 genes highly abundant in cluster A were selected (Figure 2B), 42 of which were identified as functionally important (Figure 2C), which included 27 cancer testis antigens (CTAs) such as *TPPP2*, *TAF7L* and *PRM1* etc., *TNFSF4* as an immunotherapy candidate gene involved in promising immunological response in carcinomas,^5^ four transcription factors (TFs) including *NEK5*,^6^ *MYT1*, *FGFR3* and *BRSK2* as known altered genes in numerous cancer types, four genes involved in the metabolic process of glycolysis, and four enzymes correlated with metastasis (Figure 2C). A large portion of these genes were important targets of CTAs. Generally, DEGs in cluster A predominantly exhibited oncogenic function, some of which were druggable targets, which could provide effective therapies in molecular aspects.

**FIGURE 2:**
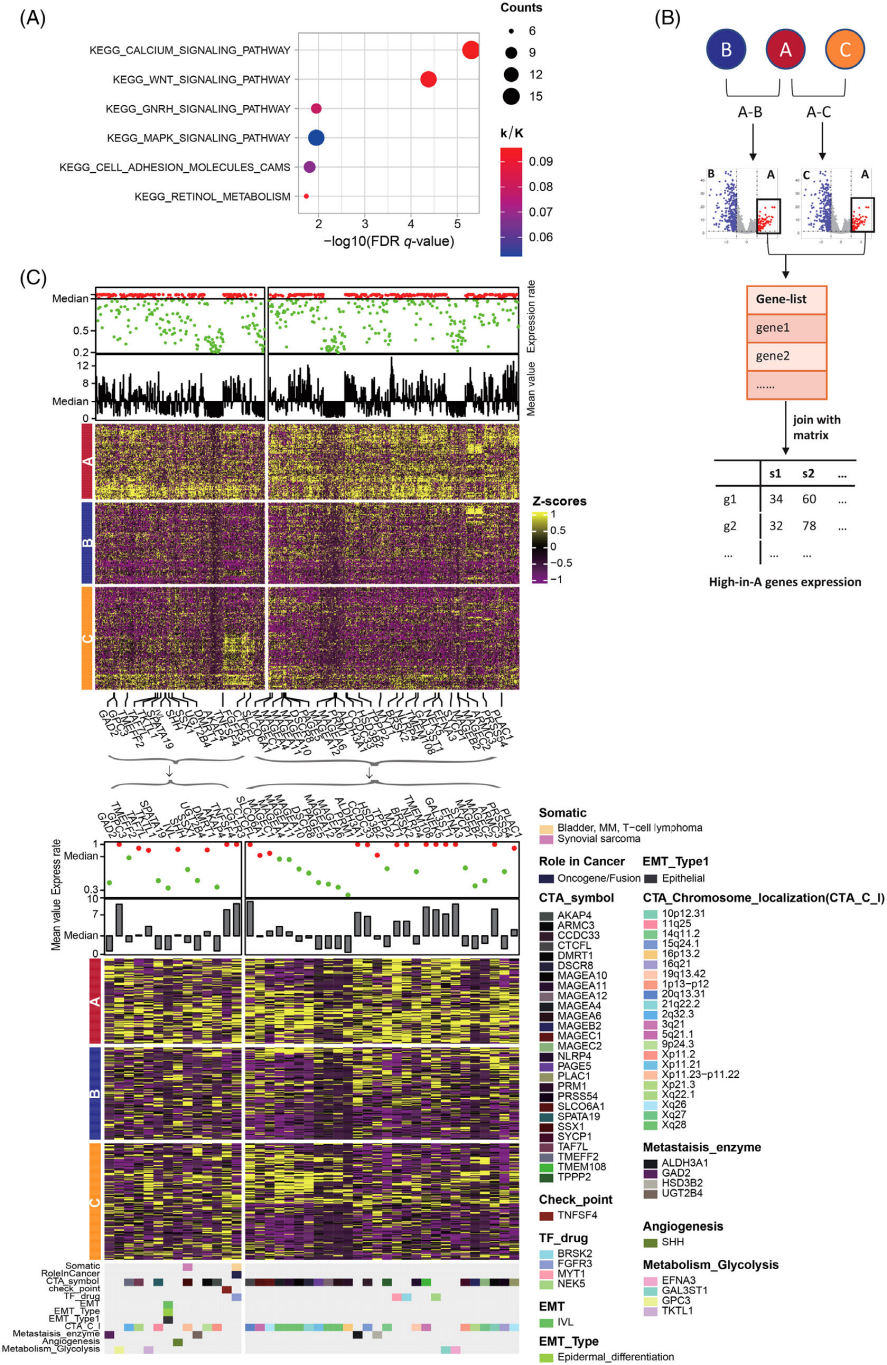
Enrichment analysis within tumor‐specific genes and biological pathways. (A) Pathway enrichment analysis identified 5 tumor‐specific KEGG pathways. (B) Schematic flow chart shows the filtering process of identifying tumor‐specific genes enriched in group A. (C) A heatmap of genes with high expression in group A. A list of 42 genes was labelled and furtherly clustered among three groups. The expression rate and mean value of each gene was calculated, distinguished and plotted by median values, and immune functions of 42 genes were elaborated and identified (Table S10).

Using similar methods, we performed analysis in OC samples of cluster B and C. A total of 43 up‐regulated Hallmark (*n* = 18) and KEGG (*n* = 25) pathways were identified as predominant immune‐related pathways (Figure 3A). And we completed a stepwise filtering process to obtain 530 genes with high expression in cluster B and C (Figure 3B), 53 of which were annotated as functionally important immune‐related genes (Figure 3C). Among of them, 17 genes (*CD70*, *LAG3*, *TIGIT*, etc.) were approved to be immune checkpoints, which could regulate the immune system.^7^ And nine genes belonged to HLA molecules, and another five genes were targeted genes for CTAs. Also, some genes like *CD74*,^8^ *ICAM1* and *PSMB9* were enrolled into the process of antigen processing and presentation, and some other genes were associated with TF correlated drugs, metabolic enzymes promoting cancer‐cell migration, and metabolic glycolysis promoting tumorigenesis. Meanwhile, MCP‐counter^9^ was applied to produce the absolute abundance scores of eight immune cells and two stromal cells in tumour samples (Figure 3D), which were significantly higher in cluster B and C. Together, our results indicated that cancer cells might reprogram OC's tumour microenvironment to promote cancer development, and immune checkpoint inhibitors may produce an effective treatment for subtype C.

**FIGURE 3:**
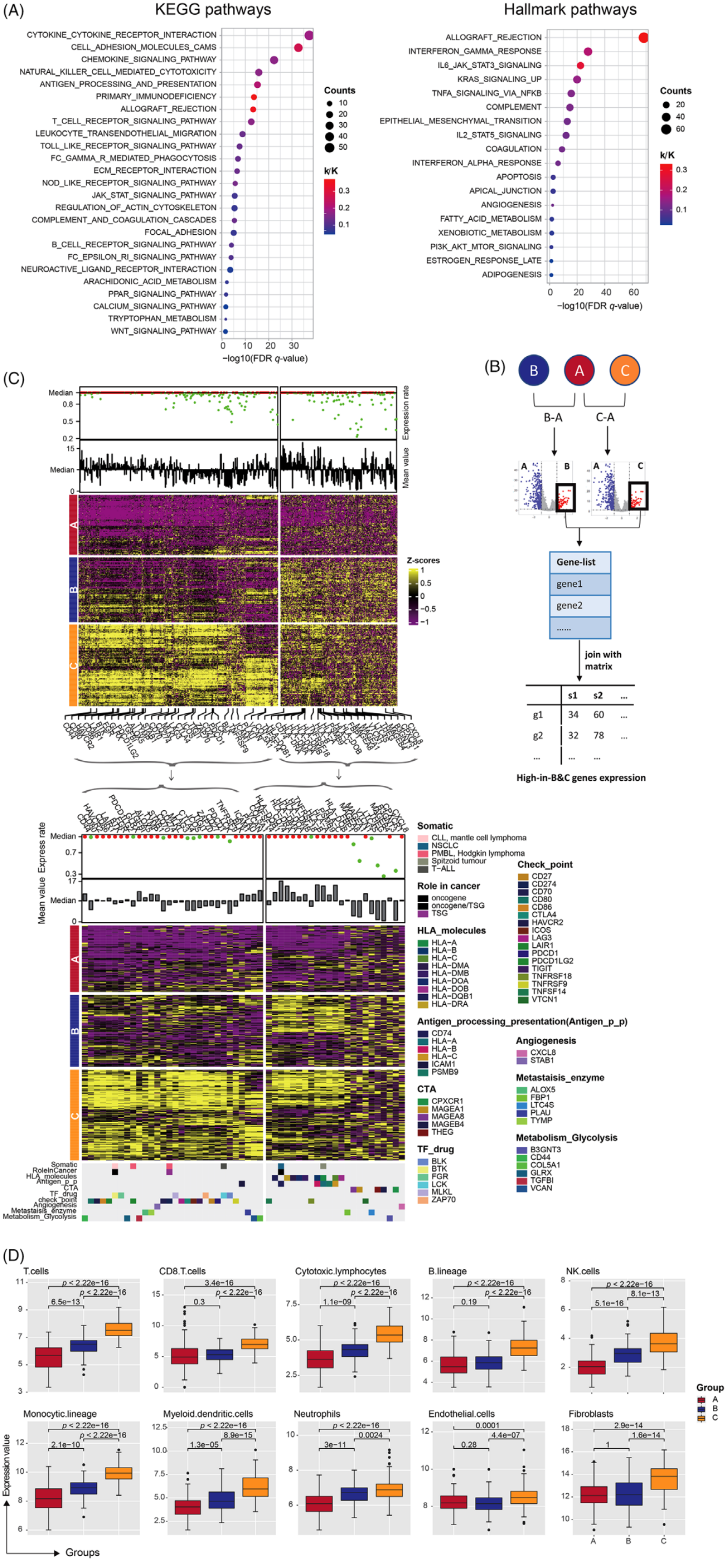
Functional enrichment and immune infiltration analysis in cluster B and C. (A) Pathways enrichment analysis identified biological pathways enriched in subgroup B and C. The curated gene sets were downloaded from the MsigDB databases. FDR *q*‐value, the *p*‐value adjusted for the false discovery rate (FDR). A *q*‐value threshold of .05 (5% FDR) is applied. The pathways are colored by their *q*‐values. (B) Schematic flow chart shows the filtering process of identifying immune‐related genes enriched in cluster B and C. (C) A heatmap of genes with high expression in cluster B and C. A list of 53 genes was labelled and furtherly clustered among three groups. The expression rate and mean value of each gene was calculated, distinguished and plotted by median values, and immune functions of 53 genes were elaborated and identified (Table S11). (D) The abundance scores of 8 immune cells and two stromal cells in three groups were visualized and significant *p*‐values between each two subgroups were labelled in plot.

Then, we performed whole‐exome sequencing analysis to filter out the most frequently altered genes (*n* = 35) on OC samples (*n* = 250). The top three genes sorted by mutation frequencies were *TP53*, *MYC* and *NDRG1*, among of which, *TP53* had a high mutation rate up to 95%, and the other two were with high amplification counts. And the amplification rate of *MYC*, *NDRG1*, *EIF3E* and *SMARCA2* was significantly higher in cluster B and C, but *NOTCH3* and *AKT2* for cluster A (*p* < .05, Figure 4A and Table S4). DNA mutation sites with missense mutation or truncating and cancer hotspots of four genes including *TP53*, *KMT2C*, *CDK12* and *BRCA2* were presented across three subgroups of OC patients (Figure 4B and Tables S5–S8). Then, we observed the performance of copy number alteration in the three groups (Figure 4C) and discovered that segment amplification of genes such as *CD27*, *KRAS* and *PTK6* were significantly more occurrence in group A on the whole, but *TP53* was with significantly more deletion in group B (Figure S3). All the integrated mutational profiles supported the genomics‐based clustering designation.

**FIGURE 4:**
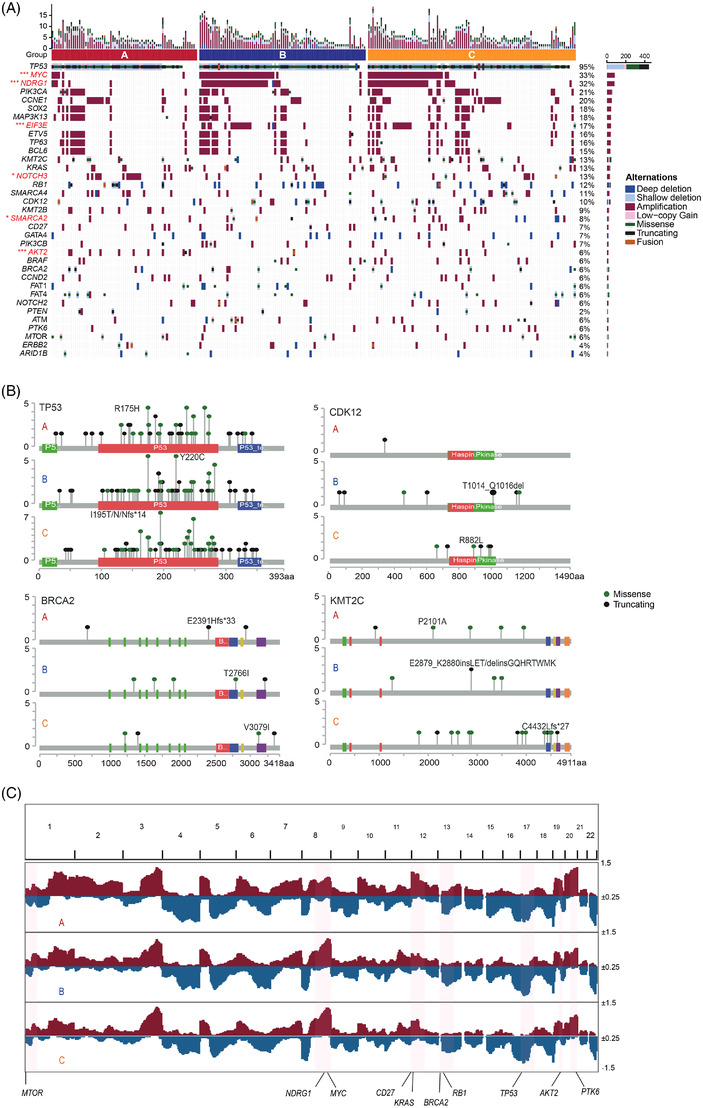
Mutation characterization among three groups. (A) The OncoPrint plot shows somatic genomic alterations identified by whole‐exome sequencing (WES). (B) Somatic mutations in four genes. Proteins domain structure of the 4 mutated genes with somatic mutations aligned. Different colors as shown in the legend indicated the functional domains and mutation types in each gene – *TP53*, *BRCA2, CDK12* and *KMT2C* genes. (C) Somatic copy number alteration (CNA) identified across 22 chromosomes by WES. The frequency of each CNA was labelled on the right axis.

Results of survival analysis and pathways enrichment revealed survival differences between Figo‐stage subgroups (≤ IIIB vs. ≥ IIIC) in immune‐enriched group, which displayed that this new classification could augment the prognostic power as an independent clinical factor (Figure S4 and Table S9). Additionally, performance of DNA methylation and protein expression among three groups was also correlated with the genomics‐based classification (Figure S5). Comparison with previous subtypes indicated the consistency in molecules and experiments with two datasets validated our clustering (Figure S6).

## CONCLUSION

Novel molecular subtypes of OC was identified with molecular heterogeneity and was useful for targeted therapy. Tumor‐enriched A can use targeted‐tumor approach while immunotherapy is suitable for group C, and group B can use a mixture of tumor killing and immunotherapy. The complex molecular landscapes of subtypes may help with precision medicine.

## CONFLICT OF INTEREST

The authors declare that they have no competing interests.

## FUNDING INFORMATION

National Natural Science Foundation of China, Grant Number: 62076015.

## Supporting information

Supporting Information

Supporting Information

Supporting Information

Supporting Information

Supporting Information

Supporting Information

Supporting Information
