# Supplementary material for: Integrative analysis identifies three molecular subsets in ovarian cancer
Source: Clin Transl Med. 2022 Sep 18;12(9):e1029. doi: 10.1002/ctm2.1029 (PMC9482804; doi:10.1002/ctm2.1029)
Supplement: Supplementary file 1 — Supporting Information [file CTM2-12-e1029-s005.pdf]

Supplementary Information-1 (Supl-1)

Patients and samples

The clinical data, mutation data, DNA methylation data, mRNA expression data and protein data were downloaded from The Cancer Genome Atlas (TCGA, https://cancergenome.nih.gov), Gene Expression Omnibus (GEO, https://www.ncbi.nlm.nih.gov/gds), The Cancer Proteome Atlas (TCPA, https://tcpaportal.org/tcpa/) and The Human Protein Atlas (HPA, https://www.proteinatlas.org/). We detected batch effects of TCGA data, results of which showed there were not batch effects (Figure S1a). And 6,194 tumor special genes were selected in GEO dataset including 20 normal samples and 25 tumor samples[1] after excluding 1,442 genes with high and medium expression in normal ovary tissue from HPA dataset. After the removal of missing values, a total of 376 ovarian cancer patients with 4,188 genes were e nrolled into this study from TCGA dataset. Figure S1b shows the average expressions and expression rates of 4,188 genes in samples. Some important genes are labeled which have high expression rates.

References

1. Lili LN, Matyunina LV, Walker LD, Benigno BB,McDonald JF. Molecular profiling predicts the existence of two functionally distinct classes of ovarian cancer stroma. Biomed Res Int, 2013. 2013:846387.

Figure-S1

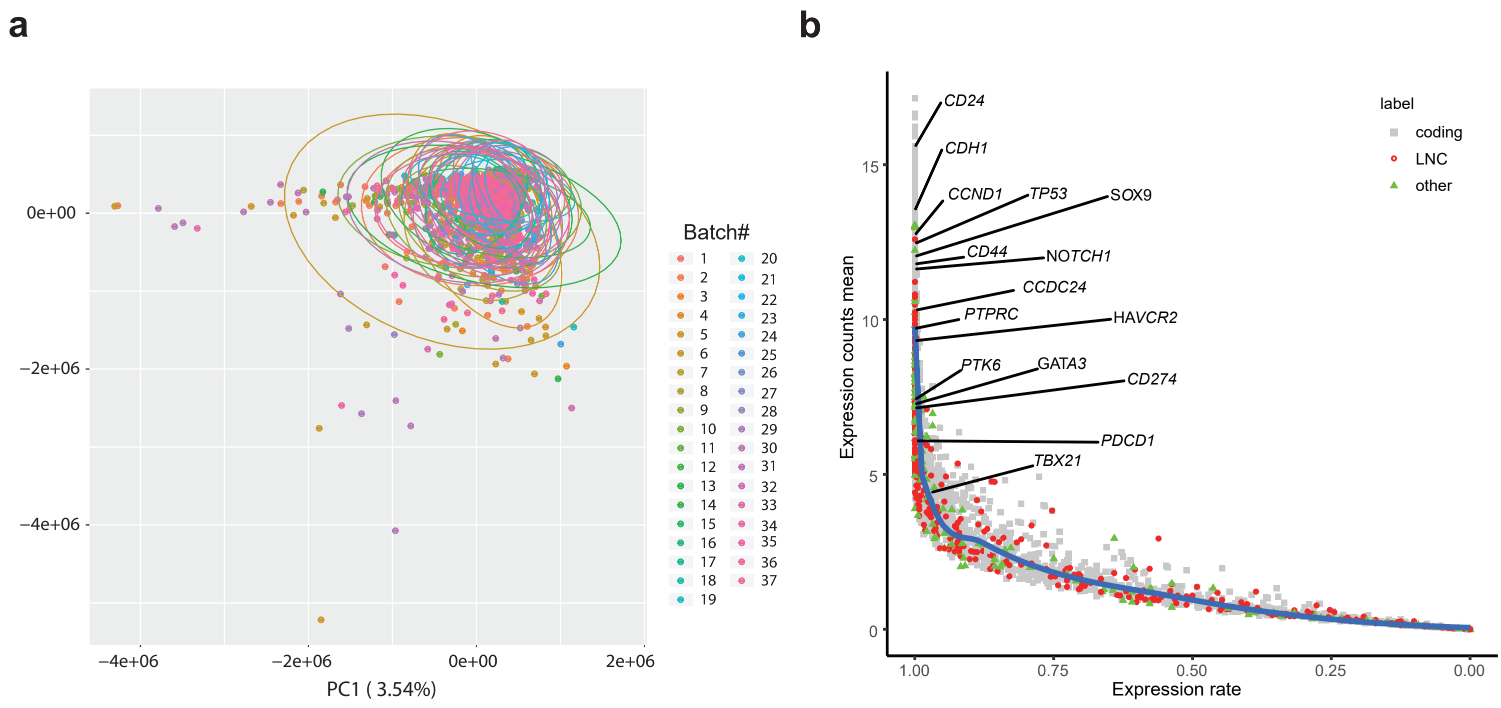

Figure S1. (a) Results of principal component analysis showed data from TCGA did not have batch effects. (b) The mean expression and expression rate of 4,188 genes. Some important genes are labeled which have high expression rates.
