# Supplementary material for: Integrative analysis identifies three molecular subsets in ovarian cancer
Source: Clin Transl Med. 2022 Sep 18;12(9):e1029. doi: 10.1002/ctm2.1029 (PMC9482804; doi:10.1002/ctm2.1029)
Supplement: Supplementary file 2 — Supporting Information [file CTM2-12-e1029-s004.pdf]

Supplementary Information-2 (Supl-2)

The confirmation about impact of immune activation and suppression molecules on survival

We found the immune cells in group C both includes immune activation and suppression molecules. To investigate its impact on survival, we found 46 immune activation molecules and 24 immune suppression molecules[1]. After joined with 1,357 different expression genes (DEGs), we got 28 genes for clustering in cluster C. From the heatmap (Figure S2a-b) we found that patients in cluster C showed higher expression (Table S3) and did not show expression differences between immune activation and suppression molecules, despite being clustered into two groups. The expression consistency of immune activation and suppression molecules in group C may make their functions mutually inhibitory and explain why survival rate did not show significance between three groups. Furthermore, we did survival analysis between these two groups in cluster C, result of which still did not reach a statistical significance (Figure S2c).

References

1. Zhou X, Du J, Liu C, et al. A Pan-Cancer Analysis of *CD161*, a Potential New Immune Checkpoint. Front Immunol, 2021. 12:688215.

Figure-S2

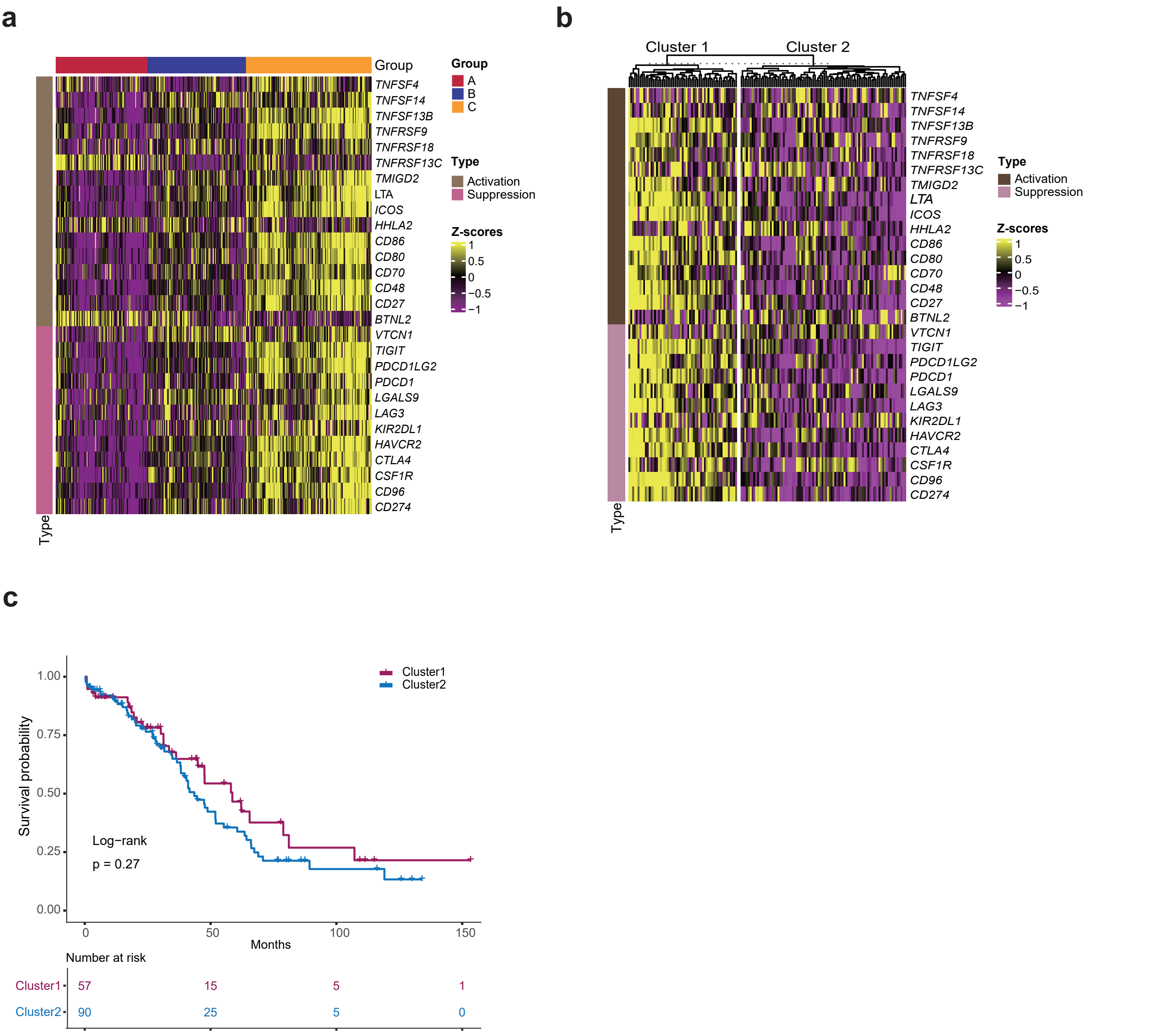

Figure S2. The confirmation about impact of immune activation and suppression molecules on survival. (a). Performance of immune cells in all samples. (b). Re-clustering of cluster C with immune activation and suppression molecules. (c). Survival analysis between two clusters in b.
