# Supplementary material for: Integrative analysis identifies three molecular subsets in ovarian cancer
Source: Clin Transl Med. 2022 Sep 18;12(9):e1029. doi: 10.1002/ctm2.1029 (PMC9482804; doi:10.1002/ctm2.1029)
Supplement: Supplementary file 3 — Supporting Information [file CTM2-12-e1029-s002.pdf]

Supplementary Information-3 (Supl-3)

***TP53* deletion**

We have calculated the numerical deletion values of *TP53* among groups according to your suggestion. *TP53* has a higher deletion rate in group B (76.25%) and C (74.00%) than group A (58.57%). The result of statistical analysis (p=0.036) also revealed the significant difference of *TP53* deletion among groups.

We also calculated the copy number values of *TP53* on each patient. The Figure S3 below shows that *TP53* is significantly deleted in group B. And deletion of *TP53* in group C is lower than that in group A, although it is not significant.

Figure-S3

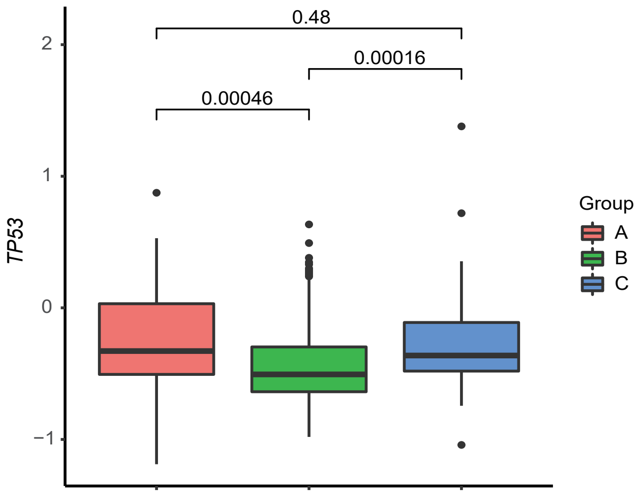

Figure S3. Deletion of *TP53* in three groups. *TP53* is significantly deleted in group B. And deletion of *TP53* in group C is lower than that in group A, although it is not significant.
