# Supplementary material for: Integrative analysis identifies three molecular subsets in ovarian cancer
Source: Clin Transl Med. 2022 Sep 18;12(9):e1029. doi: 10.1002/ctm2.1029 (PMC9482804; doi:10.1002/ctm2.1029)
Supplement: Supplementary file 4 — Supporting Information [file CTM2-12-e1029-s007.pdf]

Supplementary Information-4 (Supl-4)

Figure-S4

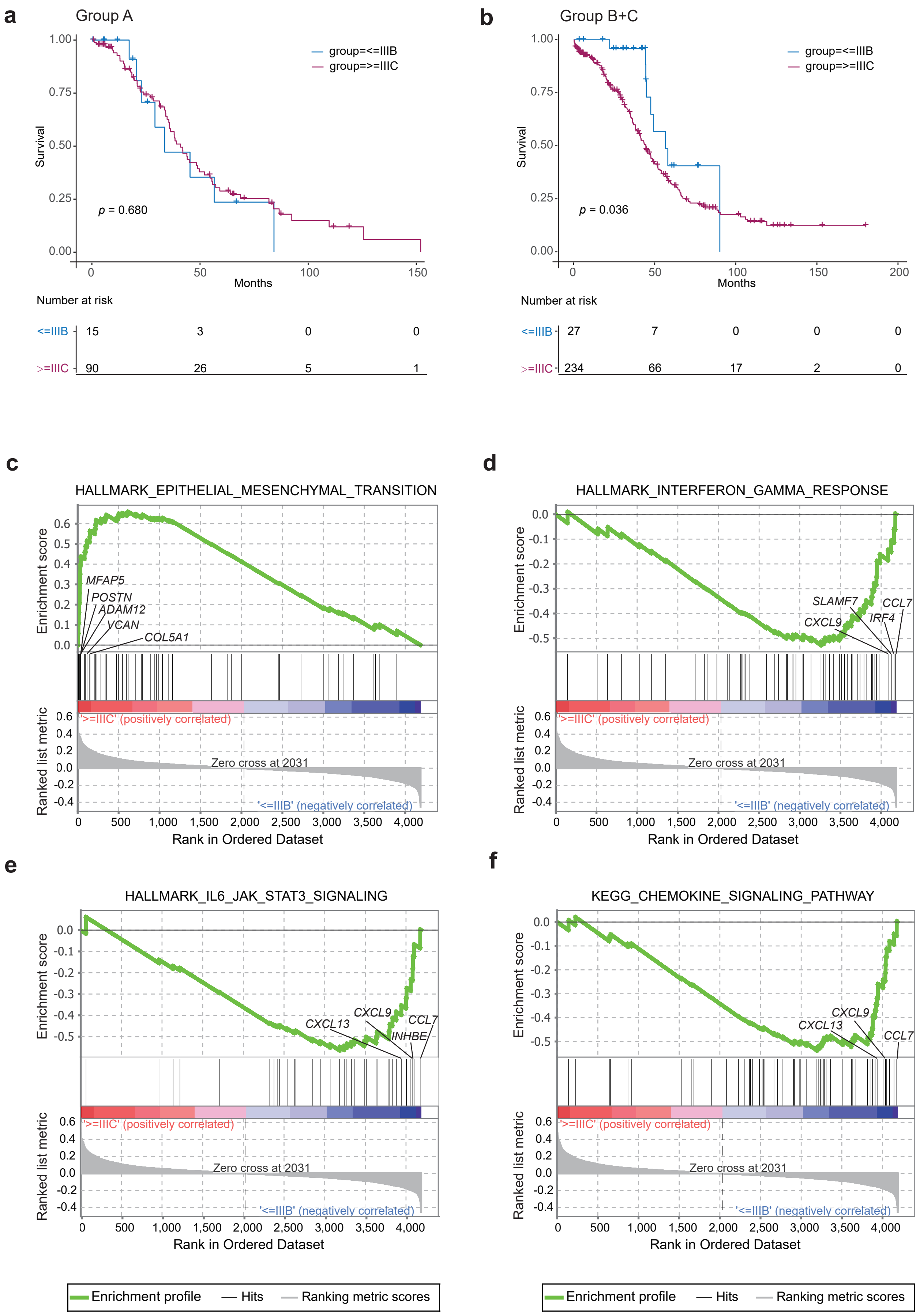

Figure S4. Survival analysis and functional performance evaluation between the new classification and Figo stage. (a-b) The Kaplan-Meier estimates showed overall survival for groups of patients in three groups after stratified as Figo stage ≤ IIIB and ≥ IIIC. P-values were computed with log-rank test. (c-f) Enrichment pathways analysis were performed between Figo stage ≤ IIIB and ≥ IIIC in group B and C. Some genes correlated with cancer development were labeled on the plots.
