# Supplementary material for: Integrative analysis identifies three molecular subsets in ovarian cancer
Source: Clin Transl Med. 2022 Sep 18;12(9):e1029. doi: 10.1002/ctm2.1029 (PMC9482804; doi:10.1002/ctm2.1029)
Supplement: Supplementary file 5 — Supporting Information [file CTM2-12-e1029-s006.pdf]

## Supplementary Information-5 (Supl-5)

**Performance of DNA methylation and protein expression among three groups**

To compare the level of DNA methylation and protein expression among three groups, unsupervised hierarchical clustering analysis was performed with DNA methylation data and proteins data (Figure S5a, S5e). We selected six genes to observe their mRNA expression and DNA methylation values and the results of correlation analysis showed negative correlations (Figure S5b-d). Analysis based on different expression proteins (DEPs, Figure S4e) displayed that cluster A was tumor-enriched group with highest expression of oncogenic markers such as P53, CDK1_pY15 and ECADHERIN but lowest expression of the immune cell marker PDL1. Similarly, cluster C showed the highest expression of the immune cell marker PDL1 but lowest expression of cancer-related markers, indicating predominance of immune cells. Cluster B, however, exhibited mixed expression features of both oncogenic and immune response and was therefore classified as "mixed group" as above. Most proteins and their encoding genes have a relatively high correlation (Figure S5f). For example, the correlative coefficients of P53 and *TP53*, PDL1 and *CD274* were 0.699, 0.496. This was in line with the classified results from the genomic analysis.

### Figure-S5

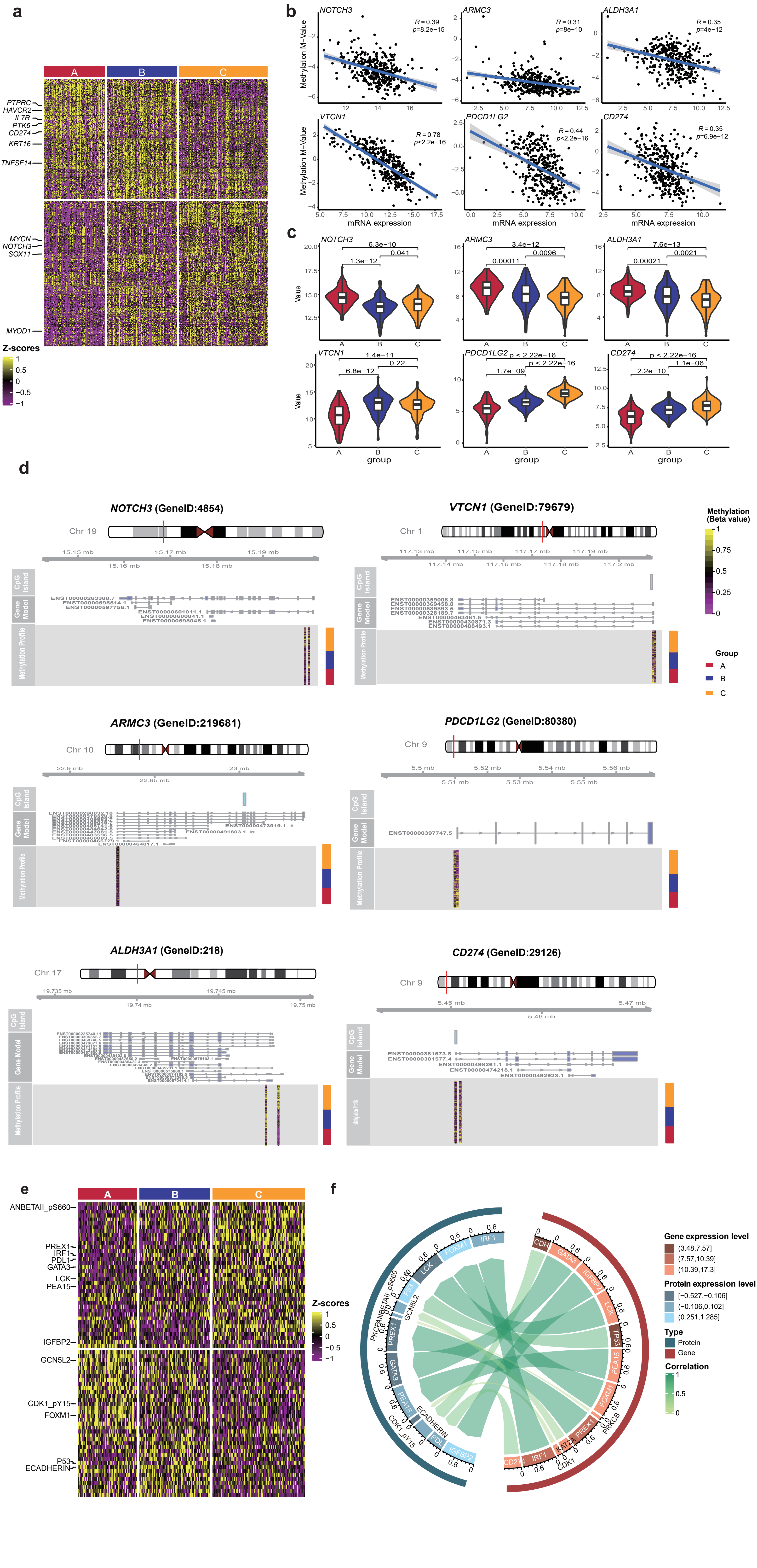


**Figure S5. Overview of the three subtypes on methylation and proteomics.** (a) Clustering of methylation data in 369 patients and 5,988 differentially expressed methylation probes. (b) The mRNA expression of six genes among three groups. (c) Correlation of six genes between DNA methylation and mRNA expression values. (d) DNA methylation of six genes on chromosome. (e) Clustering of protein expression data in 260 patients and 224 proteins. Some genes correlated with cancer development were labeled on the plot. (f) Correlation analysis between DEGs and their encoding proteins.
