# Supplementary material for: Integrative analysis identifies three molecular subsets in ovarian cancer
Source: Clin Transl Med. 2022 Sep 18;12(9):e1029. doi: 10.1002/ctm2.1029 (PMC9482804; doi:10.1002/ctm2.1029)
Supplement: Supplementary file 6 — Supporting Information [file CTM2-12-e1029-s003.pdf]

Supplementary Information-6 (Supl-6)

Validation

The validation datesets consisting of 490 samples were collected between March 1st, 2021 and April 20th, 2021 from GSE17260[1] and GSE140082[2] data sets. Patients in both datasets can be divided into three groups and the expression of seven gene markers is consistent with our grouping.(Figure S6a-b)

Comparison with previous molecular subtypes

Tothill et.al[3] idntified six molecular subtypes of ovarian cancer, subtype C1 (high stromal response), C2 (high immune signature), C4 (low stromal response), and C5 (mesenchymal, low immune signature) of which were almost high-grade serous cancer. We compared the genes highly expressed in C1, C2, C4 and C5 with genes highly expressed in each subtype of our classification. Results showed that our tumor-enriched group A had a large overlap with C5 while group C overlapped with C1 and C2, and group B distributed in C2 and C4, which revealed that our new classification corresponds with their finds. (Figure S6c) Tan et.al[4] identified five molecular subtypes Epi-A (epithelia like a, Differentiated), Epi-B (epithelia like b, immunoreactive), Mes (mesenchymal), Stem-A (stem like a, proliferated) and Stem-b (stem like b). Correspondingly, our group C had a large overlap with Epi-B and MES while group A overlapped with Stem-A, and group B had some overlap with Epi-B and Mes. (FigureS6d) Tan et.al had compared their results with Tothill's, and our results are also consistent with that.[3]

The above description validates the consistency in molecules. In addition, our classification takes a new perspective and shows great differences between tumor and immune enrichment. Furthermore, besides genomic analysis, we did a multi-platform analysis, which revealed the biological pathway of enrichment and remarkably, novel druggable targets (cancer testis antigens, enzymes, and transcription factors) that could be selected to develop effective therapies and/or tumor stratifications. And S omatic mutation analysis revealed markers as significant mutation frequencies across three subgroups.

References

1. Yoshihara K, Tajima A, Yahata T, et al. Gene expression profile for predicting survival in advanced-stage serous ovarian cancer across two independent datasets. PLoS One, 2010. 5(3):e9615.
2. Kommoss S, Winterhoff B, Oberg AL, et al. Bevacizumab May Differentially Improve Ovarian Cancer Outcome in Patients with Proliferative and Mesenchymal Molecular Subtypes. Clin Cancer Res, 2017. 23(14):3794-3801.
3. Tothill RW, Tinker AV, George J, et al. Novel molecular subtypes of serous and endometrioid ovarian cancer linked to clinical outcome. Clin Cancer Res, 2008. 14(16):5198-208.
4. Tan TZ, Miow QH, Huang RY, et al. Functional genomics identifies five distinct molecular subtypes with clinical relevance and pathways for growth control in epithelial ovarian cancer. EMBO Mol Med, 2013. 5(7):1051-66.

Figure-S6

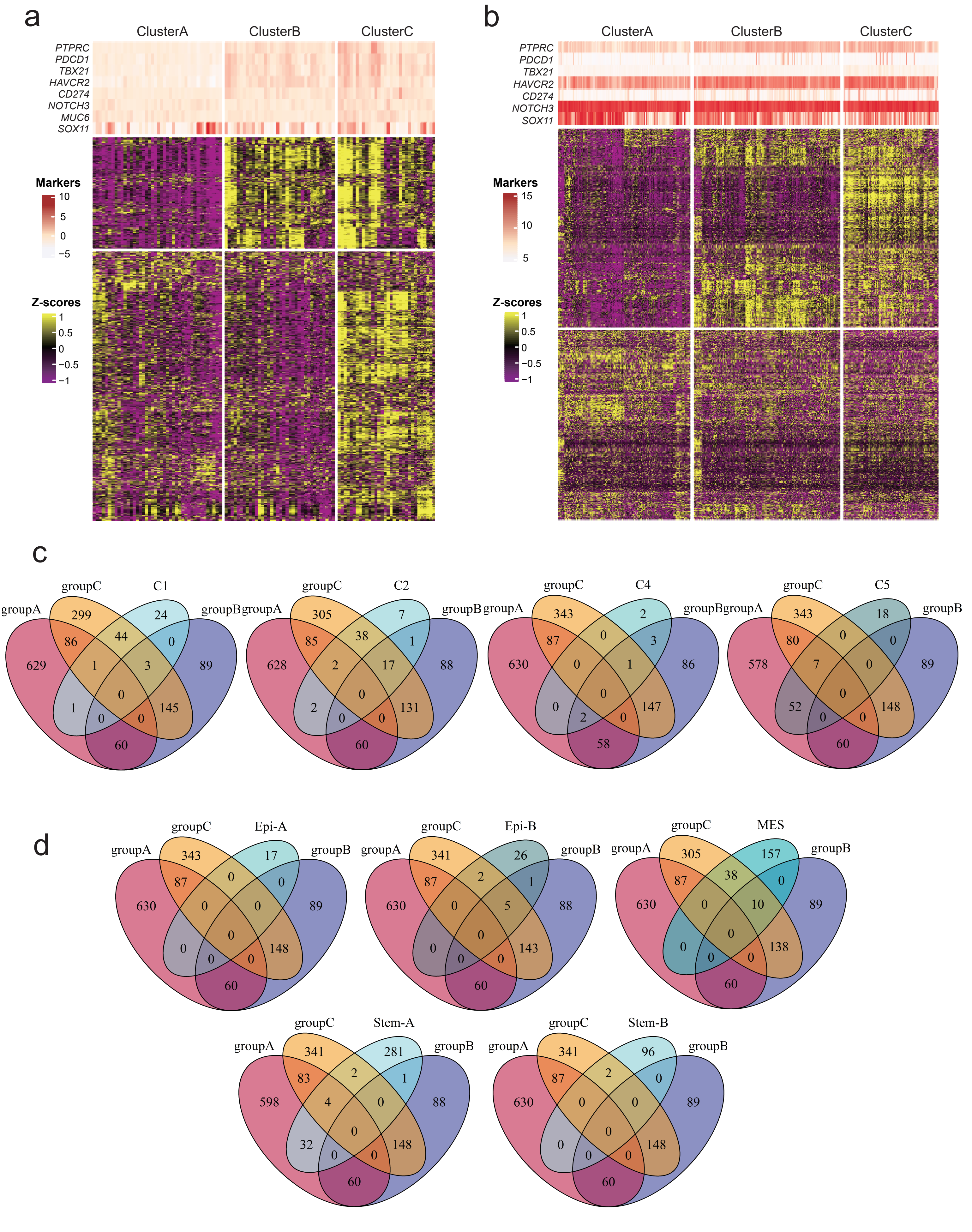

Figure S6. Validation of clustering and comparison with previous subtypes. (a). Validation with GSE17260. (b). Validation with GSE140082. (c). Gene overlaps of each group with Tothill's subtypes. (d). Gene overlaps of each group with Tan's subtypes.
